# Comprehensive characterization and valorization potential of Amazonian Sacha inchi (*Plukenetia volubilis* L.) seeds, oil, and oilcake by-products for sustainable food applications

**DOI:** 10.3389/fnut.2025.1597300

**Published:** 2025-05-21

**Authors:** Fernando E. Alejandro Ruiz, Julio F. Ortega Jácome, José R. Mora, Andrea C. Landázuri, Paola Vásconez Duchicela, Julio Vásconez Espinoza, Pablo Beltrán-Ayala, María J. Andrade-Cuvi, José M. Alvarez-Suarez

**Affiliations:** ^1^Laboratorio de Investigación en Ingeniería en Alimentos (LabInAli), Departamento de Ingeniería en Alimentos, Colegio de Ciencias e Ingenierías, Universidad San Francisco de Quito USFQ, Quito, Ecuador; ^2^Departamento de Ingeniería Química, Colegio de Ciencias e Ingenierías, Universidad San Francisco de Quito USFQ, Quito, Ecuador; ^3^Applied Circular Engineering & Simulation Group (GICAS), Chemical Engineering Department, Universidad San Francisco de Quito USFQ, Quito, Ecuador; ^4^IBioMed, Universidad San Francisco de Quito USFQ, Quito, Ecuador; ^5^Institute for Energy and Materials, Universidad San Francisco de Quito USFQ, Quito, Ecuador; ^6^Instituto de Investigaciones Biológicas y Ambientales (Biósfera), Universidad San Francisco de Quito USFQ, Quito, Ecuador; ^7^Instituto Superior Universitario Oriente ITSO, Joya de Los Sachas, Orellana, Ecuador; ^8^School of Economics, Universidad San Francisco de Quito USFQ, Quito, Ecuador; ^9^Laboratorio de Bioexploración, Colegio de Ciencias Biológicas y Ambientales, Universidad San Francisco de Quito USFQ, Quito, Ecuador

**Keywords:** Sacha inchi, functional food, baking applications, plant-based omega-3 oil source, wheat flour substitute

## Abstract

**Background:**

Sacha inchi (*Plukenetia volubilis* L.), a native oilseed from the Amazon region, has attracted increasing attention for its nutritional richness and potential applications in functional foods. However, detailed studies on the characterization and valorization of its seeds, oil, and defatted oilcake by-products from the Ecuadorian Amazon remain scarce. This study evaluates the bromatological, chemical, bioactive, and techno-functional properties of Sacha inchi seeds and their by-products from the Ecuadorian Amazon to assess their potential as functional ingredients in bakery applications.

**Methods:**

Bromatological composition and color of Sacha inchi seeds and oilcake flour were determined. Mineral content was analyzed using inductively coupled plasma optical emission spectrometry (ICP-OES). Techno-functional properties-including swelling capacity (SC), water absorption capacity (WHC), water retention capacity (WRC), and oil holding capacity (OHC)-were assessed in oilcake flour. Total polyphenol content and antioxidant capacity (DPPH and FRAP assays) were evaluated. The oil was analyzed for physicochemical quality and fatty acid composition using gas chromatography-mass spectrometry (GC-MS).

**Results:**

Sacha inchi seeds were primarily composed of fat, followed by protein. In contrast, the defatted oilcake flour showed increased protein content, notable fiber levels, and high mineral concentrations-particularly calcium (2,634 mg/kg), magnesium (3,692 mg/kg), potassium (7,684 mg/kg), and phosphorus (9,140 mg/kg). Polyphenol content (50.53 mg GAEq/100 g) and antioxidant capacity (DPPH: 36.08 mM TEq/g FW; FRAP: 62.52 mM TEq/g FW) were largely retained after oil extraction. The oil was rich in essential fatty acids, notably α-linolenic acid (46.92%) and linoleic acid (38.09%), though its oxidative instability requires attention. Techno-functional evaluation of the oilcake flour showed moderate swelling capacity (3.47 ± 0.12), WHC (4.16 ± 0.63), WRC (3.50 ± 0.24), and low OHC (1.02 ± 0.05), which may affect hydration, freshness, and texture in bakery formulations. Overall, the oilcake flour outperformed wheat flour in protein, fiber, and mineral content, supporting its potential in developing nutritionally enhanced baked goods.

**Conclusions:**

Sacha inchi oil and its defatted oilcake flour show high nutritional quality and functional potential. Their incorporation into food formulations could promote the development of healthier, sustainable products aligned with circular economy principles.

## 1 Introduction

The development of novel food ingredients that address both nutritional challenges and sustainability goals has become a central priority in food science and technology. As consumers increasingly demand foods that are healthier, minimally processed, and environmentally responsible, the exploration of alternative plant-based ingredients has gained momentum. In parallel, global food systems are undergoing a transformation to become more resilient and diversified, with emphasis on circular bio-economy models, waste valorization, and the use of local biodiversity (1). One promising strategy involves the revalorization of agro-industrial by-products and underutilized crops into functional food components with added value (2). A particularly active area of research focuses on the development of functional flours from plant-based sources, especially those derived from residues such as oilseed cakes or press meals. These flours are not only rich in proteins, fibers, and micronutrients but also exhibit valuable techno-functional properties—such as water and oil absorption, swelling capacity, and emulsifying ability—that make them attractive for food formulation (3). Their use may improve the structural, rheological, and sensory characteristics of food products, while also enhancing shelf life and nutritional quality. Despite their potential, many plant-based residues remain insufficiently characterized and underutilized, especially in the context of their application in thermally processed foods.

In particular, traditional baking, heavily reliant on wheat flour, is facing growing scrutiny due to its nutritional limitations, allergenic potential, and environmental footprint. Excessive wheat consumption has been linked to gluten sensitivity, celiac disease, and other food intolerances (4). Moreover, the large-scale cultivation of wheat contributes to soil degradation, biodiversity loss, and greenhouse gas emissions. As a result, there is increasing interest in alternative flours derived from pseudo-cereals, legumes, and oilseeds, which can diversify baked goods while improving their nutritional profile and lowering environmental impact (5). These flours not only provide essential nutrients, such as plant-based proteins, dietary fiber, and minerals, but may also confer functional benefits that influence product structure, texture, and consumer acceptance (6). While their incorporation presents technical challenges, particularly in terms of sensory quality and processing behavior, ongoing innovation continues to expand their application potential.

Among oilseed crops, Sacha inchi (*Plukenetia volubilis* L.), a plant native to the Amazon Basin, is emerging as a promising candidate for ingredient innovation. Traditionally cultivated by Indigenous communities for its nutritional and medicinal value, Sacha inchi seeds are rich in oil (up to 60%) and protein (~30%) (7). The oil is particularly valued for its high concentration of polyunsaturated fatty acids, especially alpha-linolenic acid (omega-3), and antioxidant compounds such as tocopherols (8). Consequently, Sacha inchi oil has gained international recognition in functional food and nutraceutical markets. However, the by-product obtained after oil extraction, known as oil cake or defatted flour, remains vastly underutilized. This nutrient-dense residue, rich in proteins, fibers, minerals, and residual phytochemicals, holds considerable promise as a sustainable and functional ingredient. Yet, scientific studies have disproportionately focused on the oil, with limited efforts to characterize the techno-functional properties, nutritional value, and antioxidant potential of the oil cake flour. Understanding these properties is essential for its potential application in food systems, particularly in bakery and pastry formulations, where flour performance directly affects product quality.

Despite growing interest in Sacha inchi as a sustainable food ingredient, significant knowledge gaps persist regarding the comprehensive valorization of its by-products, particularly the oil cake flour. While previous studies have examined isolated aspects of Sacha inchi seeds or oil, few have conducted integrated analyses of both the extracted oil and residual cake from the same biomass source, especially from Ecuadorian Amazon crops. The novelty of this work lies in its holistic approach: simultaneously characterizing the bromatological, nutritional, chemical, and techno-functional properties of Sacha inchi seeds, oil, and oil cake flour from a circular bio economy perspective. By examining both primary products and by-products, this study uniquely addresses the interdependence between oil extraction and the quality of resulting flours, while providing critical insights for bakery applications. Therefore, this study aims to conduct a comprehensive assessment of the bromatological characteristics, nutritional composition, chemical properties, and functional potential of Sacha inchi seeds and their defatted cake derived from local Ecuadorian Amazon crops. The investigation includes bromatological analysis, determination of macro- and microelement content, total polyphenols, and antioxidant activity in both seeds and the defatted cake. Additionally, the techno-functional properties of the oil cake flour, including swelling capacity, water absorption capacity, water retention capacity, and oil holding capacity, are evaluated. The composition and quality of the extracted oil are also characterized in terms of its fatty acid profile. This integrated approach provides valuable data to support the sustainable valorization of Sacha inchi by-products and their potential application as functional ingredients in bakery formulations.

## 2 Materials and methods

### 2.1 Materials

**Figure 1:**
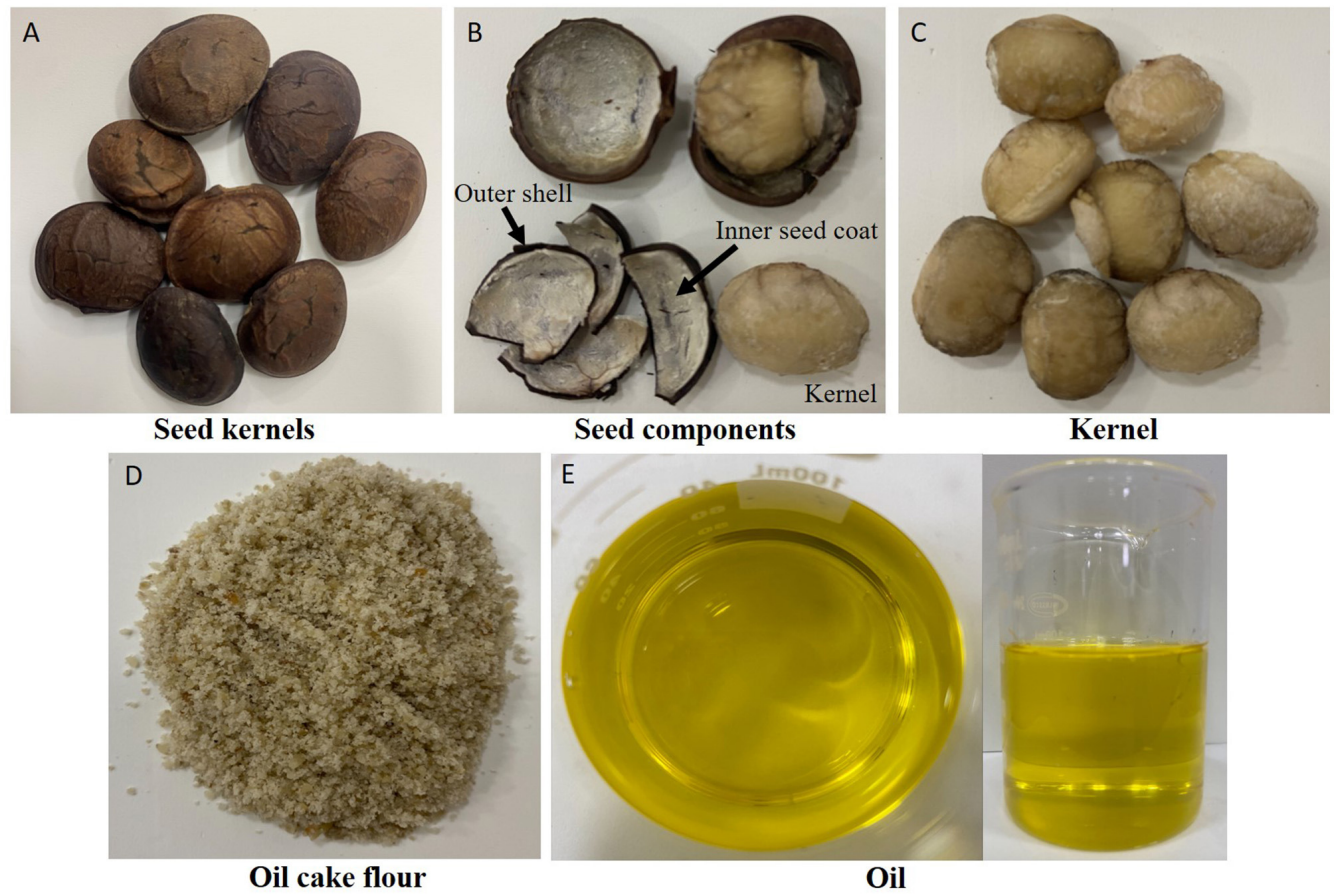
Morphological structure and processed derivatives of Sacha inchi (*P. volubilis* L.) seeds. **(A)** Whole Sacha inchi seed. **(B)** Seed components showing the kernel enclosed within a dark brown hard shell and an inner soft white tissue lining. **(C)** Sacha inchi kernel after removal of the outer shell, employed in oil extraction. **(D)** Sacha inchi oil cake flour (defatted kernel ground to a fine powder). **(E)** Sacha inchi oil (obtained by pressing the dehulled kernels).

The sacha inchi (*Plukenetia volubilis* L.) seeds, oil cake (residual non-lipid mass after oil extraction), and oil were donated by members of the Shushufindi community, Sucumbíos Province located in the northern Amazon region of Ecuador (0.1883°S, 76.36422°W). Morphologically, the seeds consist of an outer hard dark brown shell that encloses an edible kernel lined by a thin white inner tissue. This kernel is the part commonly used for oil extraction and flour production (9) (Figure 1). The raw materials were collected on three separate occasions. This community is situated on an extensive plain along the right bank of the Shushufindi River, at an altitude of 260 meters above sea level, and experiences a tropical rainy climate with an average temperature of 27°C.

After cultivation, the seeds were mechanically shelled using a seed sheller, ensuring the complete removal of the seed coat. The peeled seeds underwent an oil extraction process by cold extrusion at room temperature, whereby the raw oil was separated from the dry non-lipid fraction (oil cake). The extracted oil was left to rest for 24 h in a sealed, light-protected container at room temperature to allow the sedimentation of suspended solids. After phase separation, the upper oil layer was filtered through linen cloth and stored in sealed green glass bottles at 4°C.

A RIRIHONG 2000A multifunction grinder (Zhejiang, China) was used to grind the oil cake and seeds into a fine powder. The resulting powder was used for chemical composition and bromatological analyses. Samples were stored at 4°C in vacuum-sealed plastic bags until further use.

**Figure 2:**
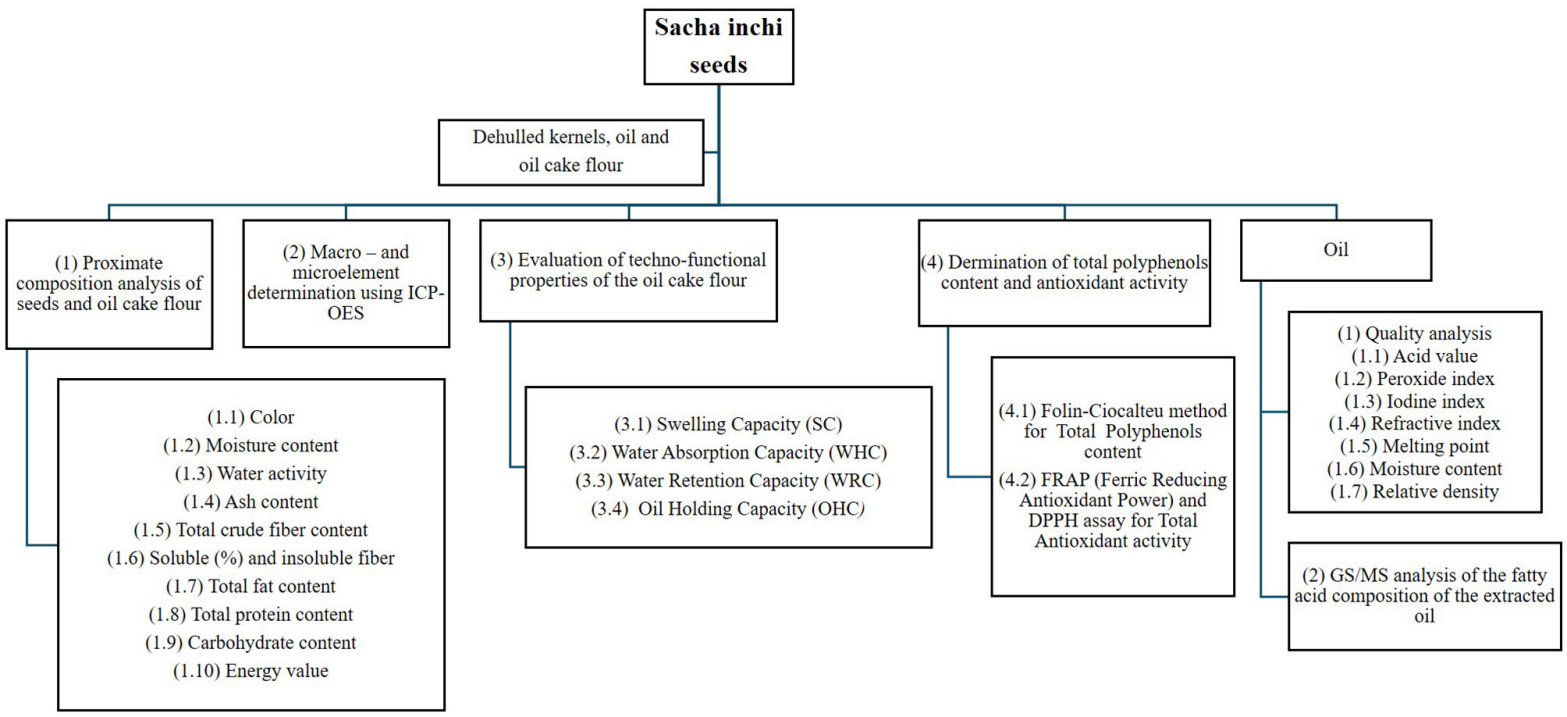
Overview of the analytical approach applied to Sacha inchi (*P. volubilis* L.) kernel, oil cake flour, and oil, including bromatological, chemical, techno-functional, and quality assessments.

The different samples, kernel and oil cake flour, were analyzed for their bromatological and chemical composition. Additionally, the sacha inchi flour was characterized in terms of its techno-functional properties and color attributes. The oil was evaluated for its quality parameters, and fatty acid profile. The overall analytical framework is outlined in Figure 2.

### 2.2 Bromatological and color analysis of Sacha inchi seeds and oil cake flour

Proximate analyses were conducted following the standardized methods of the AOAC Official Methods of Analysis. The moisture content (%) was determined gravimetrically (10), while water activity (a_*w*_) was measured using a HygroLab C1 water activity meter (Rotronic, Grindelstrasse, CH-Switzerland). Ash content (%) was determined by drying and incinerating the weighed samples (~3 g each) in a muffle at 550 ± 1°C overnight (11). The total crude fiber content (%) was determined using method AOAC 978.10, a sequential acid and alkali extractions (12), as well as the fraction corresponding to soluble (%) and insoluble fiber (%) using methods proposed by the Cereals & Grains Association (13) and AOAC (14). Total fat content (%) was determined by Soxhlet extraction using petroleum ether (15), while protein content (%) was determined by the Kjeldahl method using a nitrogen-to-protein conversion factor of 5.46 for oilseeds (16). The carbohydrate content (g/100 g) and nutritional value (Kcal/100 g) were determined using Equations 1, 2 (17), respectively, as shown below:


(1) $$\begin{array}{l} \text{Carbohydrate }(\text{g/100g})\;=\text{ 100 }-\;(\text{fat + protein}\\ \;+\text{ water + ash}) \end{array}$$

(2) $$\begin{array}{l} \text{Energy value }\left(\text{kcal}/100\text{ g}\right)\;=\;\left(\text{fat x }9\right)\;+\;\left(\text{carbohydrate x }4\right)\\ \;+\;\left(\text{protein x }4\right) \end{array}$$

Color measurements were performed using a Konica Minolta CR-400 colorimeter (Konica Minolta Sensing Americas, Inc., NY, USA), previously calibrated with a standard white tile (L^*^ = 97.65, a^*^ = −0.11, b^*^ = +1.91). Color was assessed in the CIELAB color space, which defines color using three coordinates: L^*^ (lightness), a^*^ (green to red), and b^*^ (blue to yellow). For the solid samples (sacha inchi kernel and oil cake flour), measurements were performed by placing the samples in Petri dishes and recording readings from six different locations to ensure sample homogeneity. All results were expressed in terms of L^*^, a^*^, and b^*^ values.

### 2.3 Inductively coupled plasma optical emission spectrometry (ICP-OES) analysis for macro- and micro-elements

Prior to the analysis, the samples were digested based on the methodology described in the AOAC Official Method 2015.1. “Heavy Metals in Food” (18). Briefly, samples (0.5 ± 0.1 g) were weighed into a digestion vessel, then 9 mL of nitric acid (TraceMetal™ Grade, Fisher Chemical™) and 0.4 mL of hydrogen peroxide (30% Fisher Chemical™) were added. The digested samples were filtered through a 0.45 μm PTFE membrane and brought to 50 mL with deionized water. A spiked sample was prepared in the same way as a form of quality control with recoveries between 84.70 and 99.96%.

The ICP-OES analysis was carried out using an ICP-OES system (iCAP™ 7400 Duo ICP-OES Spectrometer, Thermo Scientific™, Germany) equipped with Thermo Scientific™ Qtegra™ Intelligent Scientific Data Solution™ (ISDS) software. Quality assurance and quality control were assessed using an ICP multi-element standard solution VIII to obtain calibration curves, and the results were expressed as mg/Kg of Fresh Weight (FW).

### 2.4 Techno-functional properties of Sacha inchi oil cake flour

#### 2.4.1 Swelling capacity (SC)

The swelling capacity was assessed based on the methodologies previously described (19, 20). A portion of ~0.2 g of the sample was precisely weighed and transferred into a 50 mL conical tube. Next, 10 mL of filtered water was added, and the mixture was allowed to hydrate for 18 h at 25°C. At the end of the hydration period, the final volume of the sample was recorded to determine its swelling capacity.

#### 2.4.2 Water absorption capacity (WHC)

The water absorption capacity was evaluated using the procedure previously (19, 20). A 0.2 g sample was carefully weighed and placed in a 50 mL conical tube, followed by the addition of 10 mL of filtered water. The sample was left to hydrate at 25°C for 18 h. After this period, the supernatant was discarded, and the hydrated residue was weighed. To determine its dry weight, the residue was freeze-dried, and the final mass was recorded.

#### 2.4.3 Water retention capacity (WRC)

To measure the water retention capacity of the Sacha inchi oil cake flour samples, the methodology previously suggested by Landines et al. and Robertson et al. (19, 20). Approximately 1 g of the sample was accurately weighed and placed in a 50 mL conical tube, after which 10 mL of filtered water was added. The sample was left to hydrate at 25°C for 18 h, after which it was subjected to centrifugation using a using a HERMLE Z206A centrifuge (HERMLE Labortechnik GmbH, Wehingen, GER) at 2,000 rpm for 30 min. Following centrifugation, the supernatant was separated, and the remaining wet residue was weighed. Subsequently, the residue was freeze-dried, and its final dry weight was recorded.

#### 2.4.4 Oil holding capacity (OHC)

The oil holding capacity was determined following the method proposed by Garau et al. and Landines et al. (20, 21). For this assessment, the samples were thoroughly mixed with sunflower oil and allowed to stand overnight at room temperature. The next day, the samples were centrifuged using a using a HERMLE Z206A centrifuge (HERMLE Labortechnik GmbH, Wehingen, GER) at 1,500 rpm for 5 min. After centrifugation, the supernatant was carefully removed, and the remaining sample was weighed to determine its oil retention capacity.

### 2.5 Determination of polyphenols content and total antioxidant activity

For the analysis of polyphenols and total antioxidant activity, a hydroalcoholic extraction was performed, following the method previously described (22). Briefly, 0.5 ± 0.01 g of previously defatted (Soxhlet extraction using petroleum ether) seed powder and oil cakes flour was extracted in 5 mL of an HCl conc/methanol/water solution (1/80:19, *v/v*). The mixture was stirred for 2 h in the dark at room temperature using a magnetic stirrer. Subsequently, the mixture was centrifuged (HERMLE Z 206 A, Wehingen, Germany) at 20°C for 10 min at 10,000 rpm, filtered through Grade 1 Whatman^®^ qualitative filter paper (Sigma Aldrich, St. Louis, MO, USA), and stored at −20°C until analysis.

Total polyphenol content (TPC) was determined spectrophotometrically using an i3 UV-VIS Spectrophotometer (Hanon Instruments, Shandong, China) using the Folin–Ciocalteu method (23). Gallic acid was used to generate the standard curve, and the results were expressed as mg of gallic acid equivalents per 100 g of fresh weight (mg GAE/100 g FW).

The total antioxidant capacity was determined in parallel using the spectrophotometric methods FRAP (Ferric Reducing Antioxidant Power) (24) and DPPH (25)using Trolox as an external standard to obtain the calibration curve. The results of both methods were expressed as mM of Trolox equivalents (TEq) per gram of fresh weight (mM TEq/g FW).

### 2.6 Quality and chemical composition analysis of Sacha inchi oil

#### 2.6.1 Physical–chemical analysis for quality

The determination of the Sacha inchi oil quality indices was carried out following the standardized methods of the AOAC Official Methods of Analysis (26). The determination of the acidity index (%) was performed following the AOAC 940.28 method (27), the peroxide index (mEq O_2_/kg) according to the AOAC 965.33 method (28), the iodine index (cg/g) according to the AOAC 993.20 method (29), the refractive index using the AOAC 921.08 method (30), the melting point (°C) using the AOAC 920.127 method (31), the water content (%) through the AOAC 984.20 method (32), and the density (g/ml) using the AOAC 920.212 method (33).

#### 2.6.2 Gas chromatography-mass spectrometry (GC-MS) analysis of fatty acid content

The fatty acid content was determined using the internal standard method as described by Ponce et al. (34) and detailed in Application Note 14103 provided by the Analytical Control System suppliers (35). For this analysis, a transesterification reaction of 0.5 ± 0.01 g of the oil sample was performed with methanol (molar ratio methanol: fat 14:1), and KOH was used as a catalyst in 1% m/m to the fat. Methyl dodecanoate (2.0 mg/mL) was used as an internal standard for the quantification process. The samples were dissolved in hexane for analysis. A Shimadzu GCMS-QP-2010 Ultra Plus with autosampler/autoinjector AOC-20i/s was employed for the GC-MS analysis. A capillary GC column DB-WAX (column thickness: 0.25 μm, length: 30.0 m, diameter: 0.25 μm) was used for the analysis. The oven configuration was set as follows: an initial temperature of 120°C with a hold time of 2 min, followed by a ramp to 190°C at 15°C/min and a hold time of 4 min, then a ramp to 240°C at 5°C/min and a hold time of 10 min, and finally a ramp to 250°C at 5°C/min and a hold time of 3 min. The injector temperature was set at 250°C, and 1 μL of the sample was injected with a split mode of 1:4. Helium was used as the carrier gas, considering the linear velocity for a column flow of 0.98. Mass detection was configured to start at a *m*/*z* of 35.00 and end at a *m*/*z* of 700.00. Individual standards (1.45–2.55 mg/L) of palmitic acid [*y* = (0.9712 ± 0.0119) × + (0.0377 ± 0.0027), *R*^2^ = 0.9930, LOD = 0.0092, LOQ = 0.0278], oleic acid [*y* = (0.9951 ± 0.0086)*x* + (0.0245 ± 0.0017), *R*^2^ = 0.9994, LOD = 0.0056, LOQ = 0.0171], and linoleic acid [*y* = (0.9315 ± 0.0144)*x* + (0.0442 ± 0.0015), *R*^2^ = 0.9982, LOD = 0.0053, LOQ = 0.0161] were used to prepare the calibration curves. The mass ratio is “*x*”, and the area ratio is “*y*”. Based on the response factor of 1:1 for the previous three fatty acids (slope of about 1), which implies that *mi*/*mt* = *Ai*/*At* (mass ratio is equal to area ratio), it was possible to quantify the other fatty acids as:


(3) $$\begin{array}{l} \%\text{Fai}=\left(Ai/At\right)x100 \end{array}$$

where % Fai is the percentage of the corresponding fatty acid “*i*”, *Ai* is the area of the given fatty acid, and *At* is the total area (*At* = ∑*Ai*). In the calibration curve equations, the mass ratio is “*x*”, and the area ratio is “*y*”. The results were expressed in grams of fatty acids per 100 g of oil (g/100 g for the oil).

### 2.7 Statistical analysis

Statistical analyses were performed using IBM^®^ SPSS^®^ Statistics for Windows version 24.0. Data between different groups were analyzed statistically using a one-way ANOVA and Tukey's *post hoc* test; *p* ≤ 0.05 was considered significant. The samples were analyzed in triplicate, and results were reported as mean ± standard deviation (SD).

## 3 Results and discussion

### 3.1 Bromatological, macro- and micro-element contents, techno-functional properties and bioactivity analysis of Sacha inchi seeds and oil cake flour

#### 3.1.1 Bromatological analysis of Sacha inchi seeds and oil cake flour

Table 1 presents the bromatological analysis and chemical composition of Sacha inchi seeds and oil cake flour. In terms of moisture, both the seed and the oil cake flour exhibited low values (< 5%). The moisture content of the seed was within the range previously reported for this raw material, which varies between 3.3% and 8.32%, depending on the drying and post-harvest conditions (36–40). Meanwhile, the water activity values were high (~0.8) for the seed. The oil cake flour had a significantly higher moisture content compared to the seed (*p* ≤ 0.05), while the seed showed a significantly greater water activity (*p* ≤ 0.05). These moisture results align with previous studies, which reported a moisture content of around 4.41% in Sacha inchi oil cake flour (20) and up to 8.32 in seeds (8). The increase in oil cake flour moisture content may be attributed to several factors: (i) the defatting process increases the exposed surface area, facilitating greater interaction with atmospheric humidity; (ii) the removal of the lipid barrier reduces the natural protection against moisture absorption; and (iii) the defatting process may alter the chemical composition of the oil cake, potentially increasing the proportion of hygroscopic components, such as proteins and carbohydrates, which attract and retain moisture (41, 42). The high protein and carbohydrate levels shown in Table 1 support this hypothesis. In contrast, the lower water activity in the oil cake flour could be explained by the fact that, despite its greater moisture absorption capacity, much of this water may be bound to hygroscopic components like proteins and carbohydrates, rendering it less available. As above mentioned, both proteins and carbohydrates are present in higher concentrations in the oil cake flour, further supporting this hypothesis.

**Table 1:** Proximate and chemical composition of Sacha inchi seeds and oil cake flour.

**Analysis**	**Seeds**	**Oil cake flour**	**Wheat flour (45)**
**Proximates**			
Moisture (%)	3.09 ± 0.02^a^	4.56 ± 0.05^b^	11.1
Water activity (*a_*w*_*)	0.81 ± 0.02^a^	0.70 ± 0.01^b^	–
Ash (%)	2.70 ± 0.04^a^	4.08 ± 0.05^b^	0.56
Protein (%) (*N* × 5.46)	28.80 ± 0.43^a^	41.73 ± 0.08^b^	12
Fat (%)	46.39 ± 0.08^a^	25.36 ± 0.11^b^	1.7
Crude fiber (%)	6.21 ± 0.53^a^	10.61 ± 0.83^b^	1
Total carbohydrate (%)	19.02 ± 0.38^a^	24.32 ± 0.08^b^	71.2
Energy value (kcal/100 g)	608.75 ± 0.42^a^	492.00 ± 0.24^b^	364
**Dietary fiber (%)**			
Soluble fiber (%)	1.20 ± 0.67^a^	1.05 ± 0.28^a^	
Insoluble fiber (%)	56.32 ± 0.71^a^	59.79 ± 2.58^b^	
**Macro and microelements (mg/Kg)**			
*Macroelements*			
Calcium (Ca)	1945.97 ± 125.75^a^	2634.01 ± 56.92^b^	220
Magnesium (Mg)	2391.49 ± 86.00^a^	3692.92 ± 34.16^b^	361
Potassium (K)	5380.90 ± 269.25^a^	7684.12 ± 266.87^b^	1,500
Sodium (Na)	29.31 ± 3.66^a^	35.66 ± 3.07^b^	20
Phosphorus (P)	6121.31 ± 443.99^a^	9140.10 ± 119.25^b^	1,340
*Microelements*			
Iron (Fe)	26.71 ± 0.68^a^	43.85 ± 7.29^b^	11.8
Zinc (Zn)	35.50 ± 0.67^a^	54.54 ± 1.92^b^	11.5
**Techno-functional properties**			
Swelling capacity (S.C.)	–	3.47 ± 0.12	
Water absorption capacity (WHC)	–	4.16 ± 0.63	
Water retention capacity (WRC)	–	3.50 ± 0.24	
The oil holding capacity (OHC)	–	1.02 ± 0. 05	
**Bioactivity**			
Total polyphenols content (mg GAEq/100 g)	53.49 ± 0.82^a^	50.53 ± 0.52^a^	
FRAP assay (mM TEq/g FW)	74.52 ± 2.30^a^	62.52 ± 1.45^b^	
DPPH assay (mM TEq/g FW)	22.81 ± 1.90^a^	36.08 ± 0.85^a^	

The results are expressed as means ± standard deviation (SD). Different letters in the same row indicate statistically significant differences for p ≤ 0.05.

In general, both the Sacha inchi seeds and oil cake flour contain adequate levels of ash, which were within the range reported for other edible seeds (43) and consistent with previous findings for these seeds (2.7–6.46 %) (36–40). Ash content is an indicator of the seed's mineral content, and a moderate ash content can be nutritionally beneficial. However, in the case of the oil cake flour, it is important to note that the values reported here are lower than those previously reported for Sacha inchi seed oil cake from other regions of Ecuador e.g., 6.21 % (20). Ash content in seeds is known to vary depending on the type of seed and other factors, such as growing conditions, soil type, and processing methods (44). This could explain the difference between our results and previously reported values. In this case, the defatting process significantly concentrated (~1.5-fold, *p* ≤ 0.05) the ash content in the oil cake flour compared to the seeds. Despite this, the ash content values remained within the range of values previously reported for other edible seeds and for this same type of seed (20, 36–40).

Consistent with previous reports, Sacha inchi seeds showed a high fat content (46.34%), followed by protein (28.80%), with fat being the main component (36–40). As expected, the oil cake flour showed a significantly (*p* ≤ 0.05) lower oil content due to the degreasing process. However, after oil extraction, the residual cake still retained 25.36% fat, suggesting that a more efficient extraction process could improve oil yield. Conversely, its protein content was significantly increased by ~1.5-fold (28.80% in seeds vs. 41.73% in oil cake flour, *p* ≤ 0.05) because of the concentration of these components in the remaining paste. This increase is consistent with previous reports on Sacha inchi oil cake flour, which highlight its high protein and lower fat content (20). Compared to wheat flour, which contains about 12 g of protein per 100 g (45) (Table 1) Sacha inchi flour derived from oil cakes provides a significantly higher protein content by up to 3.5 times more. This increase is relevant from a nutritional standpoint, given that one of the suggested uses for this by-product is as a substitute for wheat flour in pastry formulations, as evaluated here.

The total carbohydrate content in Sacha inchi oil cake flour increased significantly (*p* ≤ 0.05) by ~1.3-fold compared to the seed (Table 1). This increase can be attributed to the defatting process, which reduces the lipid content and consequently concentrates other components, such as proteins and carbohydrates. Additionally, the removal of the lipid fraction may result in carbohydrates comprising a higher proportion of the cake's total composition. Compared to another study's reported total carbohydrate values in Sacha inchi oil cakes flour from Ecuador (46%) (20), the values observed here are relatively lower. However, this difference could be attributed to the residual fat content retained in the cake in this study, which reached up to 25%. In contrast, the fat content in the seeds is in line with what has been previously reported by other authors (36–40). Regarding the energy value, this decreased significantly after defatting (*p* ≤ 0.05). The oil cake flour exhibited a reduction in energy of ~0.81 times compared to the seed. This decrease is consistent with the removal of the lipid fraction, as lipids are the primary contributor to caloric intake in foods due to their high energy content (~9 kcal/g). As the fat content in the cake decreases, the total energy value is reduced, although other components, such as proteins and carbohydrates, remain in higher proportions. Closely related to carbohydrate content, the crude fiber content in the Sacha inchi oil cake flour and seed showed differences (Table 1). In both cases, the crude fiber content was high (between 6% and 10.6%), emphasizing the significant contribution of this component by both Sacha inchi seeds and oil cake flour. The crude fiber content increased significantly (*p* ≤ 0.05), by ~1.7-fold from the seed to the oil cake flour. This increase can be attributed to the removal of lipids, which concentrates other components, including dietary fiber. Notably, more than 55% of the dietary fiber in both the seed and the oil cake flour consisted of insoluble fiber, which plays a crucial role in intestinal health by promoting regular bowel movements and preventing constipation. Insoluble fiber also contributes to fecal bulk and helps maintain gut microbiota balance, enhancing overall digestive health. Compared to conventional wheat flour, commonly used in bakery products, which contain ~1% crude fiber (46) (Table 1), Sacha inchi cake flour has a significantly higher fiber content, ~10-fold greater. This underscores the markedly superior fiber content of Sacha inchi cake flour, making it an even more promising alternative to wheat flour in terms of nutritional value. This distinction is significant from a health perspective, as a diet rich in fiber, particularly insoluble fiber, has been linked to a reduced risk of gastrointestinal disorders, including diverticulosis and colorectal diseases. The high fiber content of Sacha inchi oil cake flour suggests its potential as a functional ingredient in food formulations aimed at improving gut health and overall wellbeing.

In addition to its nutritional composition, the physical attributes of flour, particularly color, play a critical role in determining its suitability for food applications and consumer acceptance. In bakery products, where visual cues strongly influence purchasing decisions, the appearance of alternative flours becomes a crucial quality parameter. Therefore, to better assess the potential of Sacha inchi oil cake flour as a substitute for wheat flour, its color characteristics were analyzed and compared with those of the native seed and conventional wheat flour. Table 2 presents the color parameters expressed as chromatic coordinates (L^*^, a^*^, b^*^, chroma [*Cr*], and hue) for Sacha inchi seed, oil cake flour, and wheat flour. The results show that Sacha inchi oil cake flour exhibited significantly (*p* ≤ 0.05) lower lightness (L^*^ = 68.55) than wheat flour (L^*^ = 91.83), indicating a darker visual appearance. This reduction in brightness is expected due to the presence of residual pigments and higher concentrations of proteins, polyphenols, and dietary fiber, which are retained in the defatted flour after oil extraction. These compounds can reduce reflectance and contribute to an opaquer and less luminous appearance. In terms of color directionality, the a^*^ and b^*^ values of Sacha inchi oil cake flour (a^*^ = 1.44; b^*^ = 19.05) suggest a light yellowish-brown hue. In contrast, wheat flour showed a nearly achromatic tone (a^*^ = −0.80) and lower b^*^ value (11.69), corresponding to a pale, neutral-white appearance (*p* ≤ 0.05). These differences are further highlighted by the chroma (Cr) values, which represent color intensity: oil cake flour showed higher chroma (Cr = 19.21) than wheat flour (Cr = 11.38) (*p* ≤ 0.05), indicating a more saturated and visually vibrant tone. The hue values, which defines the dominant wavelength of perceived color, was 85.62 for the oil cake flour, placing it between the seed (80.77) and wheat flour (94.06). This intermediate hue indicates a perceptual shift toward a more yellow tone after oil extraction, potentially due to the partial removal of reddish-brown pigments and concentration of yellow-tinted phenolic compounds or Maillard-derived products formed during cold pressing. These changes in color parameters are consistent with those observed in other oilseed flours, where mechanical processing and lipid removal often result in darker and more saturated tones (47). From a technological perspective, this darker color may influence the visual attributes of final baked goods. While it may be considered undesirable in products where a light crumb is preferred (e.g., white bread), it could be advantageous in wholegrain-style, fiber-enriched, or clean-label formulations, where a darker color is often associated with higher nutritional value and more natural or less refined ingredients. Moreover, the distinct chromatic profile of Sacha inchi oil cake flour could serve as a functional differentiator in product development, enabling the creation of baked products with unique visual identity. From a sensory and marketing standpoint, this may appeal to health-conscious consumers who associate darker, more intense coloration with artisanal or nutrient-dense foods.

**Table 2:** Color expressed as chromatic coordinates (L^*^, a^*^, b^*^, C^*^, and Hue) of Sacha inchi seed and oil cake flour.

**Color parameters**	**Seeds**	**Oil cake flour**	**Wheat flour**
*L* ^*^	71.44 ± 2.71^a^	68.55 ± 1.74^a^	91.83 ± 0.61^b^
*a* ^*^	3.54 ± 0.35^a^	1.44 ± 0.27^b^	−0.80 ± 0.18^c^
*b* ^*^	21.96 ± 1.36^a^	19.05 ± 0.85^a^	11.69 ± 0.21^b^
Cr	22.25 ± 1.29^a^	19.21 ± 0.91^a^	11.38 ± 0.31^b^
Hue	80.77 ± 1.45^a^	85.62 ± 0.92^b^	94.06 ± 1.04^c^

The results are expressed as means ± standard deviation (SD). Different letters in the same row indicate statistically significant differences for *p* ≤ 0.05. The asterisk in the L^*^, a^*^, b^*^ color nomenclature refers to the use of the CIELAB or CIE L^*^a^*^b^*^ system, a color model defined in 1976 by the Commission Internationale de l'Éclairage (CIE), which establishes an internationally recognized system for the measurement and description of color.

In summary, the color attributes of Sacha inchi oil cake flour highlight its distinctiveness and relevance as a functional ingredient. While its darker appearance may require adjustments in formulation or consumer education strategies, it simultaneously offers opportunities for innovation in the development of visually differentiated, health-oriented bakery products.

#### 3.1.2 Macro- and micro-element contents in Sacha inchi seeds and oil cake flour

The data also show that Sacha inchi oil cake flour is a good source of essential minerals (Table 1). A significant increase (*p* ≤ 0.05) compared to the seeds was observed in the concentrations of calcium (2,634 mg/kg), magnesium (3,692 mg/kg), potassium (7,684 mg/kg), and phosphorus (9,140 mg/kg) in the oil cake flour. This could be attributed to the concentration of these elements after removing part of the lipid fraction. Despite this, these results are also in line with previous studies on Sacha inchi seeds that reported high levels of potassium, magnesium, and calcium (37). Compared to wheat flour, which contains ~220 mg/kg of calcium and 1,340 mg/kg of phosphorus (45) (Table 1), Sacha inchi oil cake flour shows markedly superior levels of all these minerals. Specifically, it provides about 12 times more calcium, up to 17 times more magnesium, nearly 6 times more potassium, and almost 7 times more phosphorus than wheat flour. These differences highlight the enhanced mineral profile of Sacha inchi oil cake flour and its potential as a functional ingredient for improving the nutritional quality of food formulations. Regarding microelements, the iron (43.85 mg/kg) and zinc (54.54 mg/kg) contents in the oil cake flour are also notably high and consistent with previously reported figures for Sacha inchi seeds (37). In contrast, conventional wheat flour (whole wheat, unenriched) contains ~11 mg/kg of iron and 12 mg/kg of zinc (45) (Table 1). This makes Sacha inchi oil cake flour a significantly richer source, about 4 times more iron and over 4.5 times more zinc, highlighting its potential utility in preventing deficiencies of these essential minerals in the diet.

#### 3.1.3 Techno-functional properties of Sacha inchi oil cake flour

The analysis of the techno-functional properties of Sacha inchi oil cake flour was conducted specifically on this by-product, derived from the seeds, due to its potential application in the food industry as a possible substitute for conventional flours. The results indicate that this flour possesses characteristics that may influence its applicability in the baking industry (Table 1). Its swelling capacity (SC: 3.47 ± 0.12) is moderate compared to previously reported values for Sacha inchi flours with lower fat content, which may affect the formation of aerated structures during baking but could contribute to a crispy texture in cookies (20). Meanwhile, the water absorption capacity (WHC: 4.16 ± 0.63) and water retention capacity (WRC: 3.50 ± 0.24) indicate that this flour retains less moisture than other flours of the same type with lower fat content, which may affect dough hydration and the freshness of the final product. In bread, this could accelerate crumb hardening, whereas in biscuits, it may enhance shelf stability and promote a firm texture. On the other hand, its oil-holding capacity (OHC: 1.02 ± 0.05) suggests that its incorporation into high-fat formulations should be carefully evaluated to avoid undesirable effects on texture and mouthfeel. Its use in baking may require blending with other flours and incorporating hydrocolloids to improve dough absorption and stability. However, in biscuits and crackers, its lower water absorption and expansion capacity can be beneficial for achieving products with desirable texture and shelf stability.

Although the fat content of this flour may affect its stability and certain functional properties, it should not be considered entirely negative. The presence of beneficial fatty acids, such as omega-3 and omega-6, offers nutritional advantages that can contribute to the development of healthier food products. These essential fatty acids play a crucial role in cardiovascular health, inflammation regulation, and overall metabolic function, making Sacha inchi flour a valuable alternative for formulating functional baked goods. This nutritional aspect will be further discussed in Section 3.2. Additionally, in the case of biscuits and crackers, a higher fat content can be advantageous, as it contributes to a more tender and crumbly texture, which is often desired in these products. Therefore, while it may be necessary to adjust the fat content for bread formulations, its presence can be beneficial in the formulation of cookies, enhancing their texture and improving their sensory attributes. Nonetheless, achieving a balance between functionality, stability, and nutritional benefits should be considered for its optimal application in baking.

#### 3.1.4 Bioactivity of Sacha inchi seeds and oil cake flour

In terms of bioactivity, which refers to the presence of compounds with beneficial biological effects, such as antioxidants, Sacha inchi oil cake flour retains significant antioxidant properties and polyphenol content. Unlike essential fatty acids, which play structural and metabolic roles, antioxidant compounds such as polyphenols and other phytochemicals contribute to reducing oxidative stress and preventing cellular damage. The polyphenol content showed no significant differences between the seeds and the oil cake flour (53.49 mg GAEq/100 g and 50.53 mg GAEq/100 g, respectively) (Table 1), suggesting that these bioactive compounds are largely preserved after oil extraction. Moreover, the content values for this group of bioactive compounds were consistent with the previously reported ranges for Sacha inchi seeds (64–80 mg GAEq/100 g) (40). Likewise, the antioxidant capacity, measured using the FRAP assay (74.52 mM TEq/g FW in seeds and 62.52 mM TEq/g FW in oil cake) and the DPPH assay (22.81 mM TEq/g FW in seeds and 36.08 mM TEq/g FW in oil cake), indicates that the oil cake flour maintains considerable antioxidant activity despite the removal of lipids. The antioxidant activity of Sacha inchi seeds has been previously reported using various *in vitro* assays, including FRAP, DPPH, ABTS, and ORAC, supporting their biological potential (48). This consistency reinforces the validity of our findings. Moreover, the confirmation of antioxidant capacity through different analytical approaches further substantiates the relevance of these seeds and their by-products as functional ingredients with promising bioactivity. However, a variation in the results was observed between both methods, with a significant decrease (*p* ≤ 0.05) in antioxidant activity in the oil cake flour compared to the seed when using the FRAP assay, while an increase was recorded with the DPPH assay. This variation in antioxidant activity observed between the FRAP and DPPH assays can be attributed to the different mechanisms by which these methods assess antioxidant capacity. The FRAP assay measures the ability of antioxidants to reduce Fe^3+^ to Fe^2+^, which primarily reflects the action of hydrophilic antioxidants, such as phenolic compounds (49). The observed decrease in FRAP values in the oil cake flour compared to the seeds suggests that a portion of these reducing agents may have been lost during oil extraction or could be associated with lipid components that were removed. On the other hand, the DPPH assay evaluates the ability of antioxidants to neutralize free radicals through hydrogen or electron donation, which can be influenced by both hydrophilic and lipophilic compounds (50). The increase in DPPH values in the oil cake flour suggests that the extraction process may have concentrated certain antioxidant compounds, particularly those with higher radical-scavenging capacity. Additionally, the removal of lipids may have facilitated better solubility and the interaction of certain phenolic compounds with the DPPH radical, enhancing their measured activity. Overall, these differences highlight the importance of considering multiple methods when assessing antioxidant activity, as each assay provides complementary information about the nature and behavior of antioxidant compounds in different matrices.

Compared to wheat flour, which has a much lower polyphenol content and limited antioxidant activity (45, 51), Sacha inchi oil cake flour offers a significant functional advantage when used as a flour base. The retention of antioxidant compounds after oil extraction suggests that incorporating Sacha inchi oil cake flour into food products, such as fortified cookies, bread, or other baked goods, could provide additional health benefits, including improved antioxidant capacity. Given the rising interest in functional foods with natural antioxidants, the incorporation of Sacha inchi oil cake into wheat-based products could enhance the products' nutritional profile while offering potential health-promoting effects, such as reducing oxidative damage and inflammation.

### 3.2 Characterization of Sacha inchi oil

#### 3.2.1 Quality analysis

According to the quality analysis results (Table 3), Sacha inchi oil exhibited a relatively high acid value (2.29 mg KOH/g) compared to refined conventional oils such as sunflower, soybean, and corn, which typically show values below 1 mg KOH/g (52). This elevated value indicates a greater presence of free fatty acids, possibly resulting from suboptimal processing or storage conditions. Notably, acid values for Sacha inchi oil reported in the literature vary widely, ranging from 1.94 to 5.90 mg KOH/g, depending on drying and extraction conditions (53). In this context, the value observed in our study falls within the expected range for this type of oil, confirming its consistency with previous data. However, it is significantly higher than that reported in studies using cold-pressed oils, such as 0.38 ± 0.02 mg KOH/g (54), suggesting that extraction methods and post-harvest handling protocols may critically influence the final acidity and overall quality of the oil. The peroxide value in our sample was 9.30 mEq O_2_/kg, remaining within acceptable limits for fresh edible oils (< 10 mEq O_2_/kg), and indicating moderate oxidative stability. This is comparable to previous findings for Sacha inchi oil, e.g., 5.8 (54), 3.30 (55) 2.15 (53) and 1.85 mEq O_2_/kg (56), and also similar to high-quality olive oil (typically ≤ 10 mEq O_2_/kg) (37), supporting its suitability for human consumption when adequately stored. The iodine value (65.39 g I_2_/100 g) reflects a moderate degree of unsaturation and aligns with previous reports (45). While it is lower than that of polyunsaturated-rich oils such as sunflower (120–140 g I_2_/100 g) and soybean (120–130 g I_2_/100 g), it is close to olive oil (~80 g I_2_/100 g), suggesting intermediate susceptibility to oxidation (52). Nonetheless, much higher iodine values have been reported for Sacha inchi oil, e.g., 104.4 (53), 187.4 (56), 192.5 (54), 193.1 (57), and even up to 198 g I_2_/100 g (36), which reflect its high content of α-linolenic and linoleic acids (see Table 3). The lower iodine value observed in our sample may be influenced by factors such as seed origin, varietal differences, agroecological conditions, degree of oil refinement, or oxidative degradation during processing or storage. These variations underscore the need to standardize production practices to maintain the nutritional and functional properties of the oil. Moreover, considering the moderate oxidative stability indicated by the acid and iodine values, special attention should be given to the susceptibility of Sacha inchi oil to lipid oxidation during storage, processing, and incorporation into food matrices. This is particularly relevant due to its high content of polyunsaturated fatty acids, which are more prone to oxidative degradation. To enhance its stability and extend shelf life, several strategies can be considered. These include the addition of natural antioxidants (such as tocopherols, polyphenol-rich plant extracts, or rosemary essential oil) (58), which have been shown to delay oxidative reactions in high-PUFA oils. Technological approaches such as microencapsulation have emerged as promising strategies to protect oils rich in omega-3,−6, and−9 fatty acids, such as Sacha inchi oil, from oxidative deterioration and to improve their stability, handling, and sensory properties for food applications. Spray drying remains one of the most commonly used methods for microcapsule production, where the formation of protein-stabilized emulsions is a critical step to ensure adequate protection, encapsulation efficiency, and controlled release behavior. The use of animal- or plant-derived proteins as emulsifiers, along with appropriate wall materials (e.g., maltodextrin or gum arabic), enables the development of microcapsules with desirable physicochemical and functional properties. These technologies are particularly relevant for the incorporation of Sacha inchi oil into functional foods requiring enhanced oxidative stability, extended shelf life, or thermal processing (59). On the other hand, the refractive index (1.48) values agrees with values previously reported for Sacha inchi oil (37, 55–57, 60) and is consistent with the typical range for vegetable oils (1.46–1.49) (52), confirming its lipid profile. The melting point (−6.67°C) indicates that the oil remains liquid at low temperatures, similar to other unsaturated oils like soybean and sunflower (52). Moisture content was low (0.07%), contributing to improved storage stability. The relative density (0.93 g/mL) also matches the expected range for conventional vegetable oils (0.91–0.93 g/mL) (50), and is consistent with previous studies on Sacha inchi oil (37, 55, 57, 60). Overall, these results suggest that Sacha inchi oil has a quality profile comparable to conventional oils, offering moderate oxidative stability but a relatively high acid value, underscoring the importance of proper processing and storage to maintain its quality. Furthermore, despite variations in extraction methods and processing conditions reported in the literature, many of its key physicochemical attributes, such as density, refractive index, and iodine value, remain within a relatively consistent range. This indicates a high degree of replicability and robustness in the oil's characteristics, which supports its potential for standardization and broader application in the food industry.

**Table 3:** Sacha inchi oil quality parameters and fatty acid composition.

**Analysis**	**Value**
**Quality parameters**	
Acid value (mg KOH/g)	2.29 ± 0.18
Peroxide index (mEq O_2_/kg)	9.30 ± 1.15
Iodine index (g I_2_/100 g)	65.39 ± 0.51
Refractive index	1.48 ± 0.01
Melting point (°C)	−6.67 ± 0.57
Moisture content (%)	0.07 ± 0.01
Relative density (g/ml)	0.93 ± 0.01
**Fatty acids composition (g/100 g)**	
Palmitic acid (C16:0)	3.62 ± 1.37
Stearic acid (C18:0)	2.72 ± 1.34
Oleic acid (C18:1)	8.64 ± 0.94
Linoleic acid (C18:2)	38.09 ± 7.12
Linolenic acid (C18:3)	46.92 ± 11.60

#### 3.2.2 Fatty acid composition of Sacha inchi oil

The fatty acid profile and composition of Sacha inchi oil are notable for their high concentration of essential polyunsaturated fatty acids (PUFAs), particularly α-linolenic acid (C18:3, ω-3) and linoleic acid (C18:2, ω-6) (Table 3), making it an outstanding plant source of omega-3. These results are consistent with previously reported findings in the literature (8, 37, 61), further highlighting the oil's superiority over traditionally used oils such as sunflower, olive, corn, and soybean, which contain minimal amounts of this essential fatty acid. Comparative data from several studies reinforce the characteristic fatty acid distribution in Sacha inchi oil, with α-linolenic acid levels consistently reported between 40.47% and 50.41%, and linoleic acid between 34.08% and 37.70%. For instance, values of 41.25% α-linolenic and 37.34% linoleic acid have been reported (53); 40.47% and 37.30% (62); 42.40% and 37.70% (38); 45.20% and 36.80% (63); 46.80% and 36.20% (64); and up to 50.41% and 34.08% (36), respectively. Saturated fatty acids, such as palmitic and stearic acid, were present in much lower proportions, typically ranging from 4.00% to 5.31% for palmitic and 2.50% to 3.40% for stearic acid across the aforementioned studies. Oleic acid content showed moderate variation, generally between 8.41% and 12.95%. This strong predominance of PUFAs, particularly the omega-3 fatty acid α-linolenic acid, confirms Sacha inchi oil's nutritional value and supports its positioning as a functional lipid source with potential health benefits, especially in cardiovascular and anti-inflammatory contexts. Moreover, the consistency of fatty acid profiles across diverse studies and geographical origins suggests a robust compositional identity, further supporting its suitability for standardization and industrial application. When compared to other vegetable oils, the advantages of Sacha inchi become even more evident. For instance, sunflower oil typically contains 48–74 g/100 g of linoleic acid (PUFA) (65), whereas Sacha inchi oil provides a more balanced profile with 38.09 g/100 g of linoleic acid and a much higher concentration of α-linolenic acid (46.92 g/100 g), offering superior anti-inflammatory and cardioprotective benefits (66). Olive oil, on the other hand, is mainly composed of oleic acid (C18:1, ω-9), a monounsaturated fatty acid (MUFA), ranging from 55–83 g/100 g (67). In contrast, Sacha inchi oil contains only 8.64 g/100 g of oleic acid, which reduces its oxidative stability and makes it less suitable for high-temperature cooking. Corn oil is also rich in linoleic acid (typically 50–60 g/100 g, PUFA) but contains almost no α-linolenic acid (68), while soybean oil provides a better omega-3 profile, with 50–55 g/100 g linoleic acid and 5–10 g/100 g α-linolenic acid (69). Nonetheless, it still falls short of the exceptionally high α-linolenic content found in Sacha inchi oil (46.92 g/100 g). In terms of saturated fat content, Sacha inchi oil is also a healthier alternative. It contains low levels of saturated fatty acids (SFAs), including 3.62 g/100 g of palmitic acid (C16:0) and 2.72 g/100 g of stearic acid (C18:0). In contrast, oils such as coconut (82–90% saturated fats, mainly lauric acid) (70), palm (≈50% saturated fats, with high palmitic acid content) (71), soybean (14–16% saturated fats, including palmitic and stearic acids) (69), and sunflower (6–15%, depending on the type) contain significantly higher levels of SFAs. Thanks to its lipid profile, rich in essential unsaturated fatty acids such as omega-3 and omega-6, and its low saturated fat content, Sacha inchi oil stands out as a highly nutritious and health-promoting alternative to many commonly used vegetable oils.

## 4 Conclusions

This study highlights the nutritional, chemical, and functional potential of Sacha inchi (*P. volubilis* L.) seeds and their by-products from the Ecuadorian Amazon, demonstrating the seeds' suitability for food applications. The high protein content, rich fiber composition, and essential minerals present in the oil cake flour position it as a promising alternative to wheat flour, particularly for functional bakery formulations. Moreover, its retention of polyphenols and antioxidant capacity suggest potential health benefits that would make it a valuable ingredient in products aimed at improving dietary quality. The extracted oil, rich in omega-3 and omega-6 fatty acids, stands out nutritionally, although its oxidative stability may require further optimization to enhance its shelf life and processing versatility. Given these characteristics, Sacha inchi oil cake flour offers significant potential for the food industry, particularly in bakery and pastry products, where it could contribute to nutritional enrichment, improved functional properties, and greater dietary diversity. Future research on its technological performance, sensory attributes, and formulation strategies will be essential to fully harness its potential and facilitate its broader adoption in commercial baked goods, supporting both sustainable food innovation and health-conscious product development.

## Data Availability

The original contributions presented in the study are included in the article/supplementary material, further inquiries can be directed to the corresponding author/s.
